# Alpha gal syndrome, a relative not absolute contraindication to the use of bovine pericardium to close an intracardiac septal defect: a case report

**DOI:** 10.1186/s13019-024-02763-2

**Published:** 2024-04-16

**Authors:** Kimi Taira, Rahul Kanade, Maroun Yammine, Henry Tannous, Sathappan Kumar

**Affiliations:** 1 Renaissance School of Medicine at Stony Brook University, Stony Brook, NY USA; 2 Department of Surgery, Division of Cardiothoracic Surgery, Stony Brook University Hospital, 101 Nicolls Rd, Stony Brook, NY 11794 USA

**Keywords:** Alpha-gal syndrome, Septal defect repair, Valve repair

## Abstract

**Background:**

Alpha-gal syndrome is an allergic condition in which individuals develop an immune-mediated hypersensitivity response when consuming red meat and its derived products. Its diagnosis is important in individuals undergoing cardiac surgery, as patients frequently require large doses of unfractionated heparin or the insertion of surgical implants, both of which are porcine or bovine in origin. There are currently no guidelines for heparin administration in alpha-gal patients, with even less knowledge regarding the long-term clinical implications of these patients after receiving bioprosthetic valve replacements or other prostheses.

**Case presentation:**

We present the case of a 31-year-old male who underwent cardiac surgery in the setting of alpha-gal syndrome for a large atrial septal defect (ASD) and mitral valve prolapse (MVP). The patient continues to do well one year after undergoing a mitral valve repair, tricuspid valve repair and an ASD closure using bovine pericardium. He sustained no adverse reaction to the use of heparin products or the presence of a bovine pericardial patch. This rare case was managed by a multidisciplinary team consisting of cardiothoracic surgery, cardiac anesthesiology, and allergy/immunology that led to an optimal outcome despite the patient’s pertinent allergic history.

**Conclusions:**

This case highlights that the use of bovine pericardium and porcine heparin to close septal defects in patients with milder forms of alpha-gal allergy can be considered if other options are not available. Further studies are warranted to investigate the long-term outcomes of such potential alpha-gal containing prostheses and heparin exposure and establish the optimal decision making algorithm and prophylactic regimen.

## Background

The oligosaccharide galactose-ɑ-1, 3-galactose (alpha-gal) is a sugar molecule found in most non-primate mammals (namely pork, beef, rabbit, lamb, and venison). Certain individuals develop sensitization of immunoglobulin E (IgE) against alpha-gal, and develop an immune-mediated hypersensitivity response when consuming red meat and its derived products [1]. Those suffering from this complex allergic reaction are referred to having alpha-gal syndrome, with symptoms ranging from itching, erythema, urticaria, or angioedema to severe anaphylaxis requiring emergency treatment and hospital admission [1]. Reactions of alpha-gal syndrome may be characterized by food allergic reactions, with typical symptom onset being three to six hours after the consumption of mammalian meat, or by reactions following the use of drugs or medical products/devices containing alpha-gal in their ingredients [2]. For example, the immediate anaphylactic reaction to intravenously administered cetuximab in individuals being treated for metastatic colorectal cancer is a well-known drug-induced reaction to alpha-gal [3].

The diagnosis of alpha-gal syndrome is particularly important in those individuals undergoing cardiac surgery. Notably, most surgical implants on the market are derived from porcine or bovine tissue, which contain alpha-gal. Previous studies suggest that sensitized patients undergoing cardiac surgery may be at risk for early valve failure [4]. Additionally, the use of cardiopulmonary bypass (CPB) for open-heart surgery requires significant doses of unfractionated heparin [5]. Thus, there exists the potential risk for a significant allergic reaction in alpha-gal patients undergoing cardiac surgery. There are currently no guidelines for heparin administration in these patients, with even less knowledge regarding the long-term clinical implications after receiving bioprosthetic valve replacements or other prostheses containing alpha-gal .

## Case presentation

We present the case of a 31-year-old male who underwent mitral valve repair, tricuspid valve repair, and atrial septal defect (ASD) closure using bovine pericardium in the setting of alpha-gal syndrome. Patient consent was obtained.

**Fig. 1:**
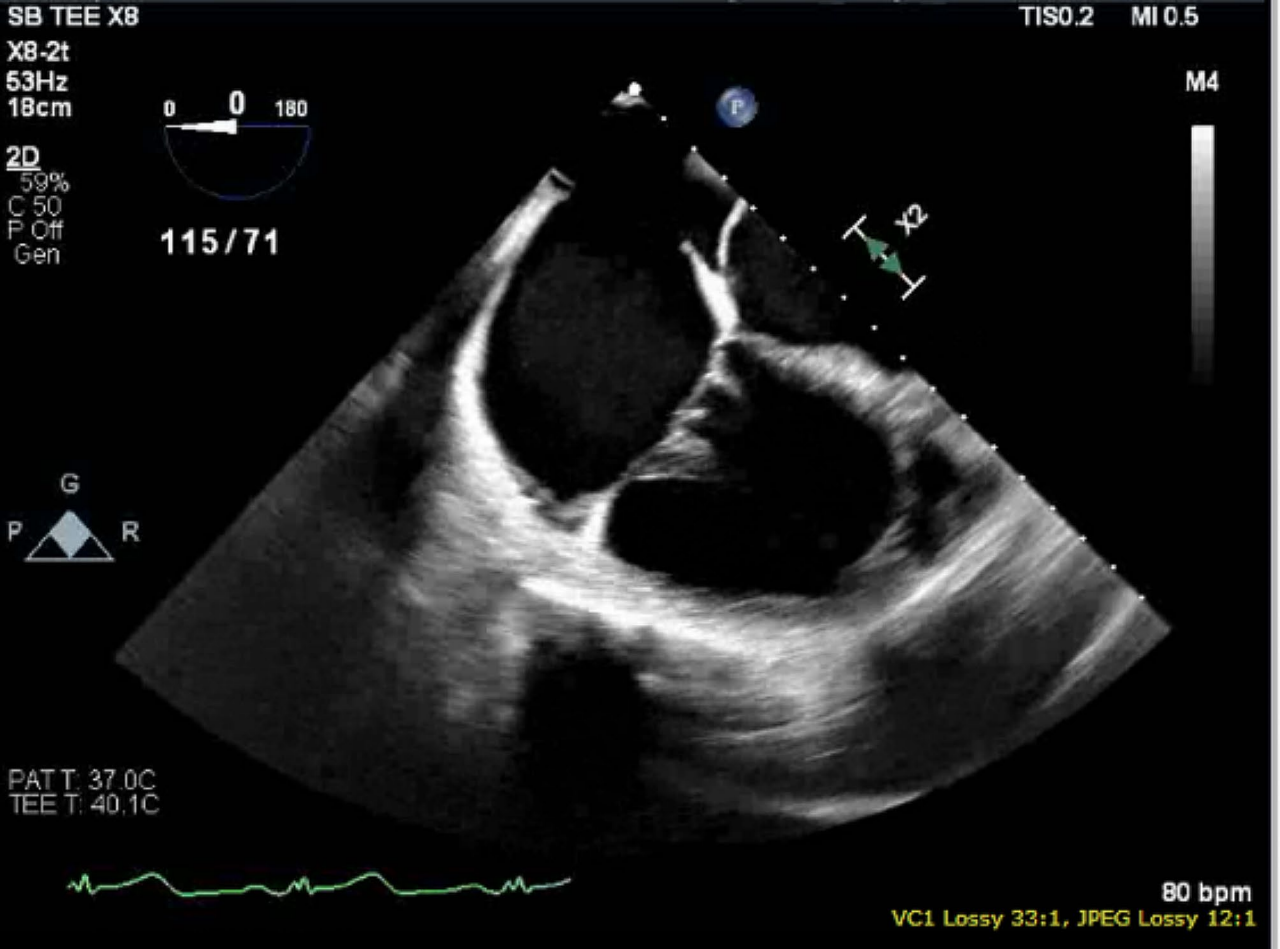
Pre-operative transesophageal echocardiogram demonstrates the presence of an atrial septal defect

The patient presented for evaluation of three months of worsening dyspnea on exertion, weakness, and fatigue. His medical history is significant for an allergy to beef and pork, lyme disease, cardiomegaly, and a heart murmur. He was admitted and underwent an extensive cardiac workup which showed severe mitral regurgitation with mitral valve prolapse and a large atrial septal defect (ASD) with dilated cardiac chambers and signs of pulmonary hypertension (Fig. 1). A CT coronary angiogram revealed no obstructive coronary disease and cardiothoracic surgery was consulted for consideration for surgical mitral valve repair and ASD closure. Operative planning for this patient included a possible replacement with a bovine or porcine prosthesis in the event that mitral repair was not possible. It also included ASD closure with a bovine pericardium in the event that the pericardium was not usable.

Additional consultation was therefore obtained from the allergy/immunology team due to the patient’s beef and pork allergy. The patient denied a history of anaphylaxis to meat. His reported symptoms following red meat consumption were pruritus and facial redness. Pre-operative serum testing included serum IgE, alpha-gal, beef, pork, and lamb IgE. The results were 182 kU/L (ref: ≤ 114 kU/L), 1.71 kU/L (ref: <0.10 kU/L), 0.62 kU/L (ref: <0.10 kU/L), 0.20 kU/L (ref: <0.10 kU/L), and < 0.10 kU/L (ref: <0.10 kU/L), respectively. To ensure that the patient was not allergic to heparin, testing was also performed on the specific heparin lot to be used during the surgery. Heparin skin prick testing was negative, as well as the intradermal testing in 1:1000, 1:100, and 1:10 concentrations. The allergy specialist therefore recommended pre-medication with diphenhydramine 50 mg, fexofenadine 360 mg, prednisone 60 mg, famotidine 40 mg, and montelukast 10 mg the night before and one hour before surgery. The patient was then taken for surgery and underwent mitral valve repair, tricuspid valve repair and ASD closure.

**Fig. 2:**
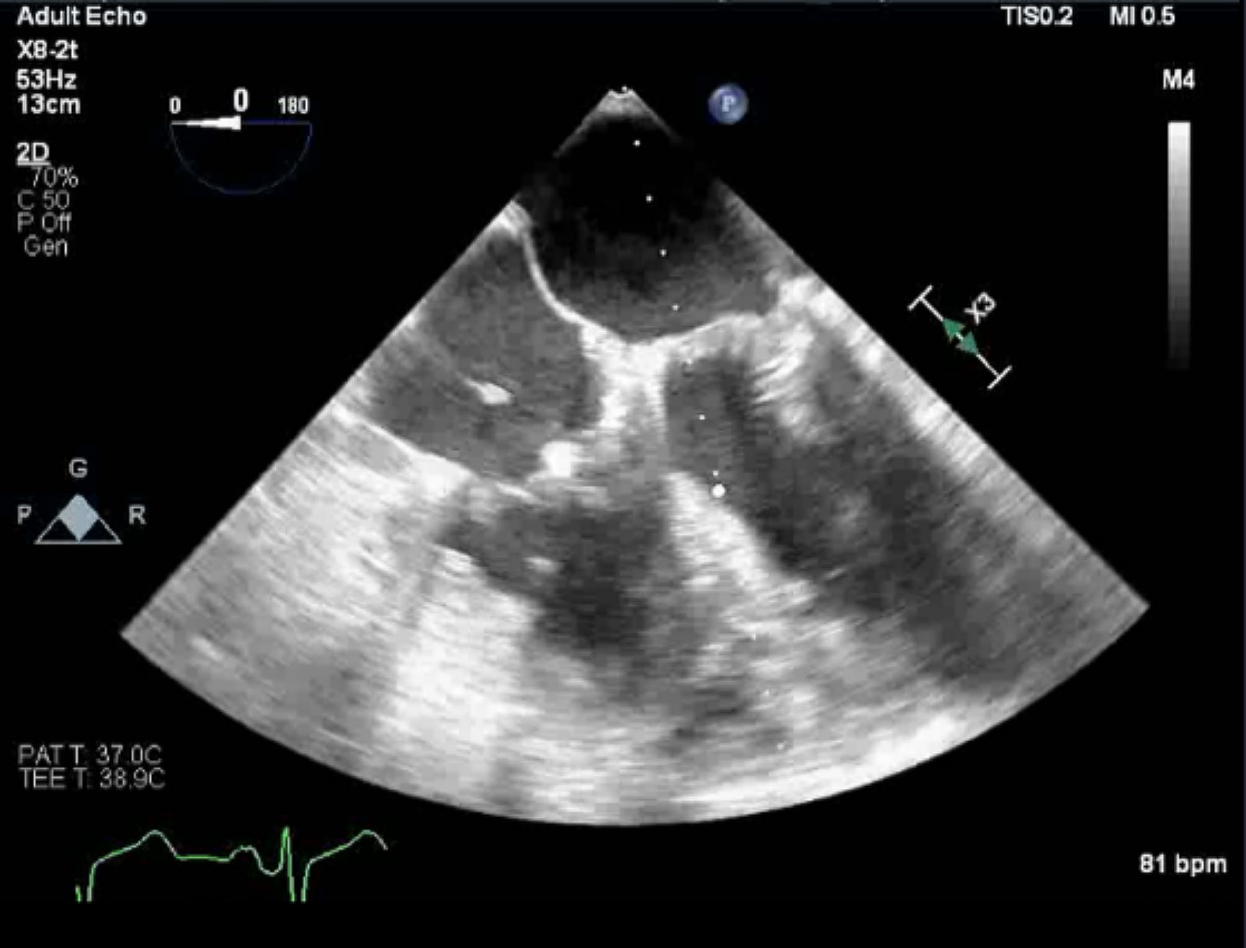
Immediate post-operative transesophageal echocardiogram shows closure of an atrial septal defect using bovine pericardial patch

During the operation and upon entry into the thoracic cavity, the patient’s own pericardium was noted to be inflamed and thickened which deemed it not usable as a patch. A decision was made to proceed with using a glutaraldehyde-fixed bovine pericardial patch to close the ASD instead, due to its availability, an adequate premedication regimen, and assessment of a clinically mild alpha-gal allergy. The patient was placed on CPB after administration of 23,000 units of heparin and an activated clotting time (ACT) greater than 480 s. The mitral valve was repaired using a 34 mm physioII annuloplasty ring (Edwards Lifesciences Corp, Irvine, CA, USA) through the opening of the large ASD. The ASD was then closed using a 3 × 2 cm bovine pericardial patch (Baxter Healthcare, Deerfield, IL, USA) and the tricuspid valve repaired using a 28 mm physio tricuspid band (Edwards Lifesciences Corp, Irvine, CA, USA). Postoperative TEE showed no residual mitral regurgitation and no flow across the atrial septum (Fig. 2). The patient was successfully weaned from CPB and his heparin was reversed with protamine.

The postoperative period was complicated by bleeding from the patient’s chest tubes requiring take back to the operating room after which he recovered well and was discharged home on postoperative day four.

**Fig. 3:**
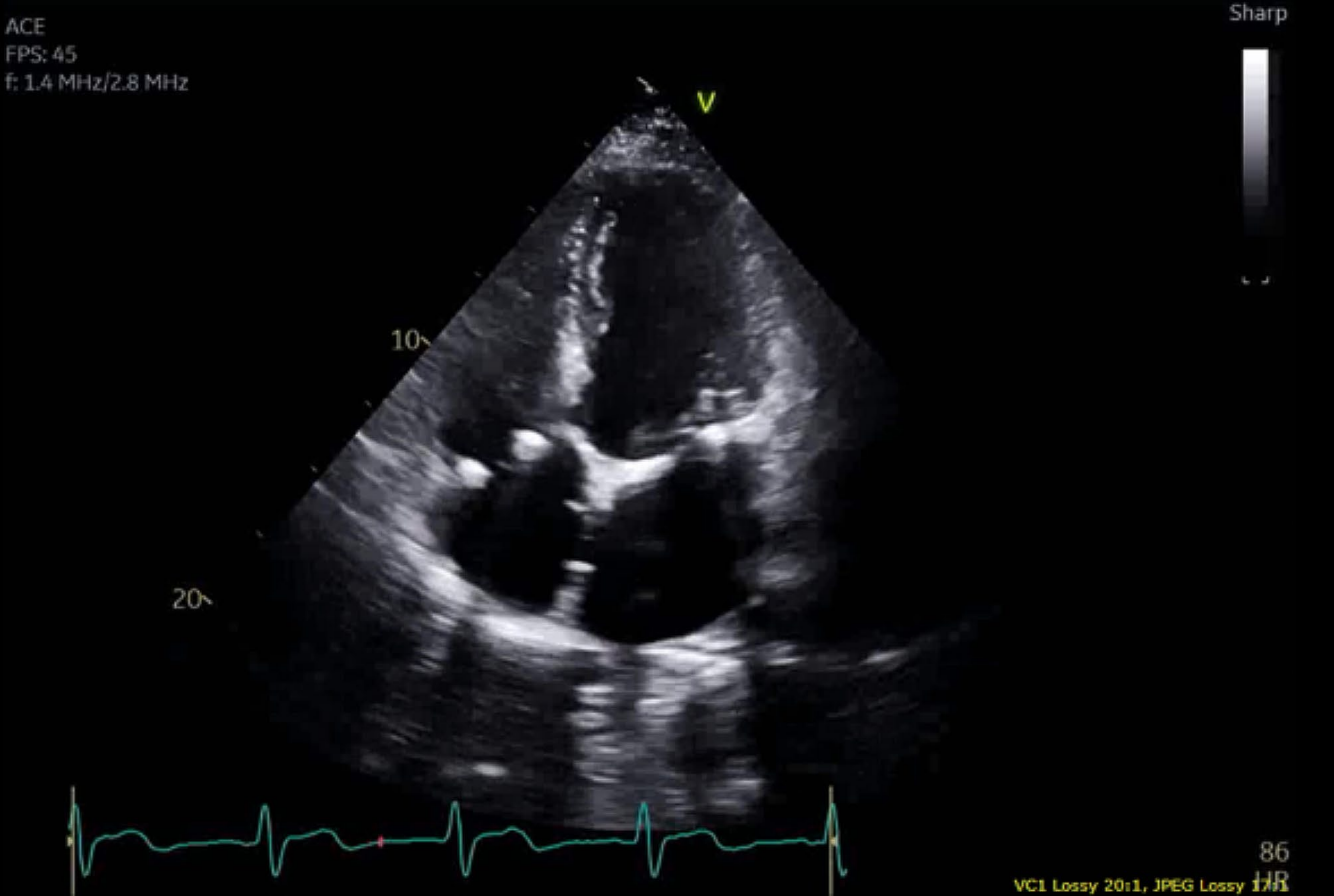
Transthoracic echocardiogram taken over one year post-operatively demonstrates an intact bovine pericardial patch

On his follow up one year following surgery, the patient continued to do well. He has not had any allergic sign or symptom and his follow-up echocardiogram revealed an improved EF, stable mitral repair with no regurgitation. The ASD patch was in an appropriate position with no evidence of dehiscence or degeneration on echo. (Fig. 3). Testing for alpha-gal IgE 15 months after surgery showed an elevated value of 7.59 kU/L.

## Discussion

Described here is a patient with an alpha-gal allergy who successfully underwent cardiac surgery necessitating the use of heparin of porcine origin, and closure of an ASD using a bovine pericardial patch. While the original approach to utilize the patient’s own pericardium was aborted due to the presence of pericardial inflammation, the patient continues to do well 15 months after surgery.

Due to the prevalence of alpha-gal-containing medications and prosthetics, individuals with alpha-gal syndrome who need cardiac surgery may be at risk for allergic reactions. As such, accurate diagnosis and preparation is key for appropriate peri-operative planning. The diagnosis of alpha-gal syndrome is based on both clinical symptoms and presence of IgE specific for alpha-gal [4]. Clinical symptoms usually occur in adult life with no previous problems after eating meat, begin 2–6 h after eating mammalian meat, and can range widely from hives to severe anaphylaxis. Alpha-gal specific IgE > 0.10 kU/L with antibodies comprising greater than 2% of the total IgE have been used as an additional diagnostic tool but this test has not been FDA approved yet. The diagnosis is usually confirmed with the improvement in patients’ symptoms when adhering to a diet avoiding red meat since there has been case description of individuals with allergic history experiencing symptoms despite a negative IgE [6].

Previous studies have reported that as many as 24% of patients who were sensitized to alpha-gal experienced severe allergic reactions to heparin during cardiac surgery [7]. This rate increased up to 50% in patients with recent IgE levels, defined as being measured within 90 days of surgery [7]. Interestingly, patients with significantly higher alpha-gal-specific IgE titers were more likely to develop a reaction, compared to those with lower levels (75 kU/L vs. 1 kU/L; *p* = .029) suggesting that increased preoperative alpha-gal titers may confer a higher risk of severe allergic reactions [7]. Our patient’s preoperative alpha-gal IgE was 1.71 kU/L, which is significantly lower than 75 kU/L. Heparin allergy was further tested with skin prick and intradermal injection of the specific heparin lot to be used during surgery which induced no allergic reaction. Heparin induced thrombocytopenia was also tested with heparin platelets antibody assay and was negative.

A previous case report described a rapid heparin desensitization protocol in a patient with a history of severe alpha-gal allergy who required cardiac surgery for multi-vessel coronary artery disease [8]. The desensitization protocol entailed initiation of twice daily oral famotidine 20 mg and cetirizine 10 mg upon admission into the cardiac surgery ICU until surgery, and consisted of infusing increasing doses of unfractionated heparin over the course of four and a half hours. After desensitization, the patient was started on maintenance dosing until he was fully heparinized for CPB. The total cumulative dose in desensitization was 1327.3 IU of unfractionated heparin. No steroids were given during the desensitization process to prevent obscuring early signs of a hypersensitivity reaction. The patient was discharged from the hospital having never experienced an allergic reaction. While this report features a successful heparin desensitization protocol, no formal guidelines currently exist and we decided to move forward with our institution’s allergy team suggestions.

In addition to the above mentioned, the decision to give heparin with premedication to our patient was favored because of the higher risks of bleeding with the other agents that cannot be reversed (such as Bivalirudin or Argatroban) which may place patients at a higher risk in need for blood and blood product transfusion as well as pericardial effusion and tamponade (9–10). The risk to benefits calculations favored the use of heparin instead of other intraoperative anticoagulation therapy especially in the presence of lower than 75 kU/L alpha-gal IgE titers and the premedication protocol suggested by our allergist.

It is also important to investigate the use of bovine or porcine surgical implants in patients with alpha-gal allergy, as these products remain permanent fixtures in the body with ongoing contact with the blood. In-vitro studies investigating the IgE response in the serum of alpha-gal syndrome patients to commercially available cardiac patches and bioprosthetic valves showed strong reactivity to the alpha-gal present on such devices, even when those devices were decellularized or glutaraldehyde fixed [4]. The true significance of these elevated antibody titers is unknown, however, given that elevated titers do not necessarily translate into clinical findings. With no association established yet between increased titers and early prosthesis degeneration, further studies are required to understand this true implication.

## Conclusion

This case highlights the potential utility of bovine bioprostheses and heparin in patients necessitating cardiac surgery who exhibit milder forms of alpha-gal allergy when other options are limited.

Further studies are warranted to investigate the long-term outcomes of the exposure to such potential alpha-gal containing prostheses and heparin, and establish the optimal decision making algorithm and prophylactic regimen for patients with alpha-gal syndrome requiring cardiac surgery.

## Data Availability

Not applicable.
